# Hybrid Blockchain and Internet-of-Things Network for Underground Structure Health Monitoring

**DOI:** 10.3390/s18124268

**Published:** 2018-12-04

**Authors:** Byung Wan Jo, Rana Muhammad Asad Khan, Yun-Sung Lee

**Affiliations:** Department of Civil and Environmental Engineering, Hanyang University, 222 Wangsimni-ro, Seongdong-gu, Seoul 04763, Korea; joycon@hanmail.net (B.W.J.); unsaboa@hanyang.ac.kr (Y.-S.L.)

**Keywords:** blockchain, smart contract, structural health monitoring (SHM), Internet-of-Things (IoT)

## Abstract

The Internet-of-things (IoT) and blockchain are growing realities of modern society, and both are rapidly transforming civilization, either separately or in combination. However, the leverage of both technologies for structural health monitoring (SHM) to enable transparent information sharing among involved parties and autonomous decision making has not yet been achieved. Therefore, this study combines IoT with blockchain-based smart contracts for SHM of underground structures to define a novel, efficient, scalable, and secure distributed network for enhancing operational safety. In this blockchain-IoT network, the characteristics of locally centralized and globally decentralized distribution have been activated by dividing them into core and edge networks. This division enhances the efficiency and scalability of the system. The proposed system was effective in simulation for autonomous monitoring and control of structures. After proper design, the decentralized blockchain networks may effectively be deployed for transparent and efficient information sharing, smart contracts-based autonomous decision making, and data security in SHM.

## 1. Introduction

The recent explosion of the Internet-of-things (IoT) is delivering innovative services in sectors such as business and industry. It has shown great applicability in fields including intelligent transportation, defense, public safety, smart cities, home automation, construction, and mining sector [1,2,3]. An IoT network is a seamless integration of heterogeneous devices with diverse functions of remote monitoring and communication with other devices. Presently, most of the IoT solutions rely on the centralized server paradigm, either connected to a main or cloud server through the internet. However, the enormous number of connected devices with limited computational power and the massive data volume generation associated with centralized IoT architecture are elevating the challenges of network bottlenecks, data security, and scalability. To solve the challenges of centralized IoT networks, adoption of a decentralized approach can be an interesting solution. Most recently, blockchain technology [4,5] has offered decentralized networks and has attracted the attention of researchers and stakeholders worldwide. Various fields in which blockchain has shown its applications are finance, healthcare, agricultural products, supply chain management, grid monitoring, and the government sector [6,7,8,9,10,11]. Blockchain helps shift the centralized IoT paradigm towards decentralization, with additional benefits of anonymity, trustworthiness, low-maintenance cost, scalability, robustness against attacks, improved user experiences, and intermediaries-free transactions. Several functionalities can easily be attained with the help of blockchain technologies that were never possible in past IoT. Leveraging blockchain with IoT can overcome the significant challenges of future IoT platforms [12].

An important domain of IoT application is structural health monitoring (SHM), in which sensors are attached to structures to sense their physical condition for ensuring operational safety and efficiency [13]. These sensors transmit data to a gateway or the main server running the algorithms for the SHM. Additionally, structures are also equipped with other sorts of sensors (actuators) to automatically respond to an emergency. SHM provides precise real-time information for assessing the health conditions, dynamic characteristics, and behavior of structures [14]. It enables earlier damage detection and eliminates the cost of routine inspections by accurately tracking the critical responses and evaluating the structures for any sign of deterioration without affecting their integrity. Most critically it improves public safety [15]. Moreover, SHM helps in allowing on-the-fly modifications (during construction), as well as improving the service quality of structures, future design considerations, and crucial decision making for maintenance planning [16,17]. IoT is providing efficient, accurate, and low-cost platforms for SHM. Moreover, few studies [18] have combined IoT SHM with cloud services to enable ubiquitous services and data analysis to access the stability of smart structures. Unfortunately, previously developed IoT–SHM systems rely on centralized network architectures, which are more prone to issues, such as data security, single point of failure, and bottleneck bandwidth. Therefore, integration of blockchain with IoT–SHM can contribute strongly to provide promising advantages, such as transparency, data security, and robustness.

### 1.1. Motivation

Usually, in a SHM program, various trustless parties (monitoring team, client, maintenance squad, and managers) are involved. The data involved is confidential and valuable raising important security issues. Monitoring data can be a lucrative target for other organizations and can be easily accessible and manipulated. Thus, there is no guarantee that the monitoring data provided is real or untampered with. In SHM, interorganizational sharing of SHM data is complex, untrusted, and less-reliable for transparency. Nonetheless, recently, many researchers [19,20] have introduced IoT-cloud based systems for SHM. Still, there is a lack of trust and transparency for interorganizational sharing of data. One reason is because comparable developed IoT-based systems for SHM are centralized (single point of failure). As a result, with the enormous growth in volume of data, these systems are becoming more prone to challenges, such as bandwidth bottlenecks, false authentications, data security and privacy, scalability, and data storage. Therefore, the central server concept should be eliminated and replaced by distributed networking. In this regard, new research horizons can be open by integrating blockchain with IoT–SHM. Maintaining data privacy (shared among all participants) on blockchain is a complicated issue [21]. This shortcoming of blockchain networks can be a key to its adoption in SHM for enabling immutable transparent cross-institution information sharing among the involved untrusted parties (client, maintenance team, and monitoring team).

### 1.2. Objectives and Our Main Contributions

Generally, in SHM, real-time monitoring and decision making have prime importance, whereas blockchain-based decentralized networks can be less efficient in terms of energy and time [22]. Therefore, this study proposes a novel hybrid architecture for decentralized data processing related to SHM using blockchain and IoT to enhance safety through autonomous decision making, data security, data storage, and cross-institutional transparent information sharing. The proposed hybrid architecture divides the blockchain network into core and edge networks, which help make the system efficient. This study leverages blockchain-based smart contracts with IoT–SHM data to create a distributed ledger of structural events as immutable transactions for a client, monitoring team, and maintenance team. This ledger keeps a full record of events, and trusted information sharing removes any point of conflict as well as automatically triggers an alert for emergency situations. The key contributions of this study are as follows:

1. We first propose blockchain–IoT-based distributed network for transparent and secure information sharing in SHM.
2. We explain the consensus mechanism along with hash function of proposed architecture.
3. We propose the use of blockchain-based smart contracts in SHM for autonomous decision making and control.
4. We place the SHM data of an underground coal mine in a blockchain–IoT network to evaluate the feasibility and performance of the proposed model based on different parameters.
5. We provide a side-by-side comparison in tabular form for state-of-the-art recent technological advancements in SHM with blockchain–IoT-based SHM network.

The rest of the paper is organized as follows. Section 2 emphasizes the applications of blockchain technology and smart contracts, and explains the status of technology adoption in SHM. Section 3 introduces the proposed system design along with the proposed model flow. Section 4 covers the proposed study model and deeply explains the designed smart contract. System analysis comparison with other studies and network performance evaluation are explained in Section 5. Discussions and limitation of the proposed system are discussed in Section 6. Finally, Section 7 concludes the study with future avenues of research.

## 2. Preliminaries and Related Work

### 2.1. Applications of Blockchain and Blockchain–IoT

This section offers a detailed literature review of blockchain applications, including technical reports, industrial applications, and governmental scale implementations. Examples include machine-to-machine (M2M) transactions, supply chain management, management and tracking of assets, IoT, health care, and tourism [23,24,25]. A private project, “Blockchain for Agri-food” [26], was started in 2017 with a special focus on the better understanding of blockchain technology application in agri-food. This project developed the proof-of-concept and established a guideline for the feasible insertion of basic information on smart contracts related to agri-food. Wang et al. [27] investigated the potential of blockchain applications in the construction industry with special attention on contract management, machinery leases, and construction supply chain management. Presently, blockchain-based commercial deployments are increasing rapidly. For instance, IBM incorporated the Hyperledger Fabric platform [28] and introduced a distributed open-source IoT–blockchain business framework. Slock.it [29,30] is part of the infrastructure for a future sharing economy. When using this platform, anything can be rented, sold, or shared with unknown parties trusted over smart contracts. This infrastructure can open and grant access to the devices carrying a suitable token or key. In this case, the owner of property sets a timed permission access after fixing a price for that property. Anyone interested can use the smartphone app to pay Ether and get permission to use an available property. A deep insight into IoT smart contracts for supporting sharing services and autonomous workflow has been explained by Christidis et al. [21]. They examined various potential blockchain–IoT areas such as the energy sector, supply chain management, tracking, billing, and e-trading. In [31], the case for groundbreaking innovations in the construction industry with the integration of IoT and blockchain was made.

### 2.2. Smart Contracts

In 1994, Nick Szabo first coined the term smart contract with the key objectives of self-executed and self-enforced transactions that follow a set of rules to make a contract [32]. The contractual clauses written in a computer program by the users to execute and upload provide a basis for blockchain smart contracts. Blockchain-based smart contracts are immutable and distributed to all the participant nodes in the network. The potential application areas for smart contracts are endless and hardly limited to cryptocurrency. In fact, smart contracts can revolutionize M2M transactions, supply chain management, asset tracking, ownership, authentication, and digital media records [33]. Comparatively, the commercial deployment of smart contracts is rapidly widening its horizons. Some other application areas of smart contracts are travel booking sites, hotels, airlines, and businesses that involve value exchange. Therefore, blockchain-based smart contracts can easily be deployed for other fields such as SHM.

### 2.3. Status of Technology in SHM

For the last few decades, SHM has enabled accurate and cost-effective monitoring of inaccessible places and large structures such as tunnels and underground structures. In [34], the authors highlighted the importance of SHM, especially with reference to underground structures. Initially, wired communication was the only reliable way for efficient SHM. In this regard, various researchers have put forward their contributions. For instance, Hisham et al. [35] monitored the strain distribution of an existing tunnel during the boring of 100 m nearby twin-tunnels. Ran et al. [36] contributed to SHM by deploying a comprehensive, long-term monitoring and safety evaluation system for the construction safety of an underground metro station.

A phase shift was observed in SHM with the invention of wireless sensor networks (WSNs). The use of WSNs allowed more reliable, cost-effective, and long-term SHM of underground structures. A hybrid system [37] of WSNs and wired communication was successfully deployed at London Underground Jubilee line for efficient and cost-effective SHM. Bennett et al. [38], reviewed the application of WSN-based SHM at Prague and London underground tunnels and highlighted critical factors, challenges, and issues. Additionally, the authors described a WSN design and well-suited topology for these structures. Another study, [39], utilized WSN for the SHM of underground train tunnels and investigated the feasibility of energy efficient WSN for this purpose. For real-time safety, during the construction of a metro tunnel in China, a web-based system for safety risk early warning was introduced [40]. This system allowed hybrid data fusion to assess safety and issue early warnings.

With the rapid development and innovation in the field of information and technology, traditional SHM was upgraded to IoT-based SHM. In this regard, recently, some studies [41,42] have been introduced focusing on the utilization of IoT and fiber Bragg grating (FBG) for real-time safety and early warnings and to enhance safety conditions at Yangtze Riverbed Metro Tunnel construction. A fiber Bragg grating-based monitoring system [43] was successfully implemented at the Zhuji coal mine. This system is based on IoT to monitor and share information in real-time. Consistently, SHM has been integrating with more and more technological advancements, resulting in the latest form of SHM comprising of WSN, IoT, and cloud computing. An extensive and elaborative review related to the opportunities and challenges of WSN with cloud computing in SHM was presented by Ruoshui and Ian [44]. Mahmud et al. [19] presented a complete IoT platform for the SHM. This system in combination uses IoT and cloud computing to run various analyses related to structural safety. Another SHM study [20] also integrated IoT and cloud computing. However, all these previously developed systems are based on a centralized architecture that makes them prone to bottleneck attacks and data security risks, as well as less reliable for transparent information sharing among all the participants [45]. Combining the blockchain technologies with IoT–SHM systems provides benefits in terms of lower operational cost, scalability, immutability, and transparent decentralized resource management. Therefore, leveraging blockchain with IoT–SHM aims to overcome the significant challenges of realizing the future decentralized SHM platforms.

## 3. System Design

SHM structures are usually equipped with various sensors (displacement, strain, and temperature) for monitoring any structural changes. In our proposed system, initially, all the monitored data from installed sensors is collected at a local gateway for pre-processing, filtering, and data arrangement. Once the formatting is complete, the data is fed to a blockchain-based smart contract to analyze the state of the structure. In Ethereum, Oracle is the most common trusted-third-party service that runs over the gateway to input blockchain-based smart contracts as well as connect smart contracts with the real world [46]. The blockchain network timestamps the gathered information, and the smart contracts are evaluated against pre-defined limits for autonomous decision making. Upon meeting the warning or alert conditions, smart contracts automatically communicate with attached devices for early warnings and concerned departments.

### 3.1. Architectural Design Overview

The proposed structure of blockchain is private and consortium-led to allow only authorized parties such as managers, monitoring team, client, and maintenance team. This helps in reducing the excessive exposure of data by requiring authentication and allowing access only to the applicants. In consortium blockchain networks, pre-approved members operate blockchain and a valid block must contain signatures from a minimum number of members. This ensures that no rogue node could insert a false transaction in the chain. Moreover, it is impossible to edit an approved smart contract; it cannot be killed but can only be replaced by a new contract.

**Figure 1:**
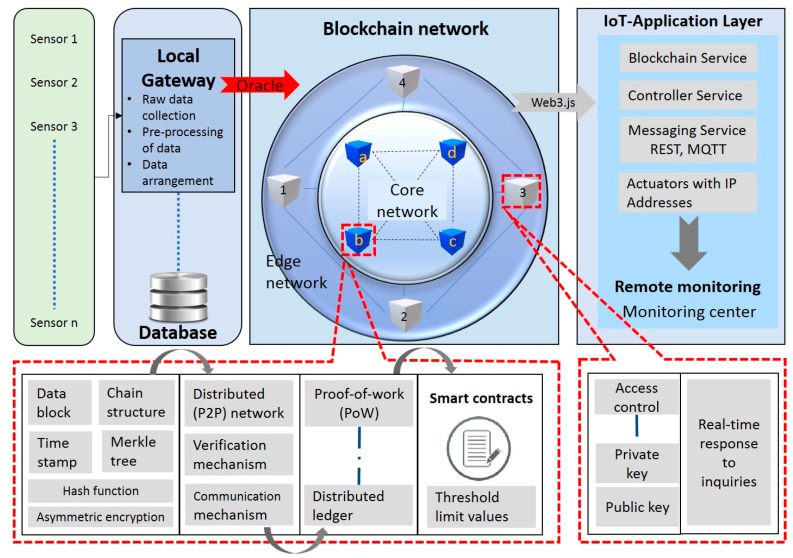
Proposed blockchain–Internet of thing (IoT) network architecture for structural health monitoring (SHM).

The present study has been designed to utilize decentralized blockchain smart contracts for efficient SHM and to share information efficiently using IoT technologies. The blockchain network in this proposed study has been divided into two sub-networks: core and edge networks, as shown in Figure 1. This architecture guarantees no confidential data will be stored on the blockchain; instead, it will record the occurrences of structural events on a distributed ledger, which can keep the track record of events in the form of transactions. This architecture has been provided with two types of storage: raw data storage at the local database and structural events storage at the core blockchain network. This architecture creates a new block in the core network of the blockchain upon successful processing of data. All the alerts and responses from smart contracts will be recorded as a completed transaction in the core blockchain network. Authentication of data in the blockchain is provided by linking this network to the SHM record. Figure 1 shows a general blockchain–IoT-based architecture for the SHM.

### 3.2. Proposed Model Workflow

In our proposed model, the edge nodes have limited storage capacity and computational power. Edge nodes serve as a centralized server for the real-time response of inquiries and offer low latency and bandwidth usage. Edge nodes hold access control mechanisms and contain the signature keys of participants. The core network comprises miner’s nodes with high storage capacity responsible for the generation of new blocks, verification of proof-of-work (PoW), and contain smart contracts for autonomous decision making. Digital signatures and immutable hash functions were deployed to ensure the integrity of data in the core network. Moreover, the core network verifies the authentication of a transaction occurring over a blockchain network. This division of the proposed model into the core network and edge network is helpful in making the system efficient to respond to queries and add resilience to external attacks.

### 3.3. PoW Scheme Algorithm

The characteristic of private blockchain networks to trust only well-defined and authenticated participants makes them secure against many vague attacks [21]. Even though in private blockchain networks, authenticated participants are not involved in mining or PoW, they are being managed by a consensus mechanism. Therefore, it is of vital importance to define a consensus mechanism for blockchain network.

Recently, the PoW has become an important consensus mechanism for blockchain networks. The PoW ensures data safety in blockchain networks by close integration, managing the trust, and verifying the transactions using hard-to-forge mathematical calculations. The proposed study for SHM based on IoT and blockchain deploys the PoW consensus mechanism along with the SHA-256 hash function. However, one of the major drawbacks of PoW as discussed is the waste of resources (electricity) [47].

To generate a block in the PoW mechanism, a miner node collects all the pending transactions and after hashing them in hashes of the Merkle tree, it iteratively hashes the collected data along with their hashes. This iterative hashing continues until the hash of transactions becomes equal to or less than a pre-determined target value serving as a threshold. This target value is the minimum number of hashes which should be performed to generate a block in the PoW mechanism. Mathematically this can be expressed as:

(1) H(n‖H(b))≤t

where *H* is the cryptographic hash function, *b* is the current block content, and *t* is the target value.

Usually, the target is the 256-bit number comprising of *k* special numbers of zeros; this makes PoW difficult and roughly requires 2^k^ attempts to solve the puzzle. In this mechanism, the determination of proof is a linear function and the lower target values demand more hashing efforts. Basically, it works by evaluating the computational forces of the participant nodes and ensures data consistency as well as consensus security [48]. PoW is a probabilistic mechanism as it changes the input of a hash to alter a hash. Ethereum utilizes the Dagger-Hashimoto function to speed up the hash’s computation. The system ensures the generation of a new hash against each iteration by repeatedly changing *nNonce* and coinbase, which ultimately alter the hash of Merkle tree root in a block header. The probability of finding *nNonce* of proof *H* for a given target *t* is given as:

(2) $$P(H\leq t)=t/2^{256}$$

Upon finding the hash, a successful miner node broadcasts the proof, input transactions, and other associated data helpful to determine *H*. Another node validates the proof by re-computing and therefore adds a new block to the blockchain. The input (*ln*) is taken as challenge with complication (*c*). Output of the mathematical computed algorithm is (N, **k, B, M**), where **k** is the index of selected leaves, **B** is the selected leave, and **M** is the Merkle tree collected proof. In this algorithm, *T* is the number of elements in the array and *Hs* is the variable-size of the hash function. A more detailed description of the PoW mechanism can be found in [49]. Algorithm 1 shows the pseudo-code of PoW.

**Algorithm 1.** PoW consensus mechanism algorithm.
**Input:***input (In)*, *complication* (*c*), *individual division*
*(d)*, *and division length* (*dl*) **Output:** (N, **k**, **B**, **M**) **Begin**Step 1**Build** input-challenge-dependent memory *Al* [1 …*T*] as *d* individual divisions of length *dl*Step 2 **Compute** root *φ* of the Merkle tree *A*Step 3 **Select** Nonce NStep 4 Compute *ɣ_O_* = H_S_ (*N|| φ* || *k*)Step 5 **For** 1 1 ≤ *j* ≤ *B*
***Do***   *i_j-1_* = *ɣ_j-1_*
*modT*   *ɣ_j_* = H_s_ (*ɣ_j-1_* || *Al* [*i_j-1_*] ± *k*)Step 6 Back sweep in reverse order *υ* = H_s_ (*ɣ_L_* ||...||*ɣ_1-lmod2_* ± *k*)Step 7 **If**
*υ* contain *c* binary leading zeros, **Then**Step 8  **return** (N, **k**, **B**, **M**)Step 9 **Goto** Step 3 **End**

## 4. Study Models and Implementation

### 4.1. SHM Data Adoption

With reference to SHM, the working of smart contracts for autonomous decision making heavily relies on the threshold limit values. In this study, to check the applicability of the proposed blockchain-IoT network, as an example, the SHM data has been taken from our previous work [50] focusing on the utilization of FBG sensing to monitor an underground mine structure and determines the “damage index of mine (DIM)”. The sensed data is initially gathered at a gateway for pre-processing and filtering. Then, the principal component analysis along with the broken stick rule determines the optimum number of components. The normalized Euclidean Ichino-Yaguchi distance measures the symbolic distances between the objects helpful for the determination of DIM. Finally, these distances are compared using cluster analysis with those of the undamaged state. Being an index, DIM has no units. A detailed mathematical explanation for the calculation of DIM is out of the scope of this paper but can be found in our previous work [50]. The following are the major reasons to select this above-mentioned study to check the applicability of the proposed blockchain and IoT based distributed network.

- The developed system is IoT-based for structural monitoring of an underground coal mine and operates efficiently under the harsh conditions of the mine;
- it provides an easy opportunity to combine the IoT–SHM system with a blockchain network;
- DIM has been clearly defined with detailed mathematical steps;
- DIM values range between 0 (undamaged) and 1 (damaged), which can be further divided into categories representing the mine structural conditions.

**Figure 2:**
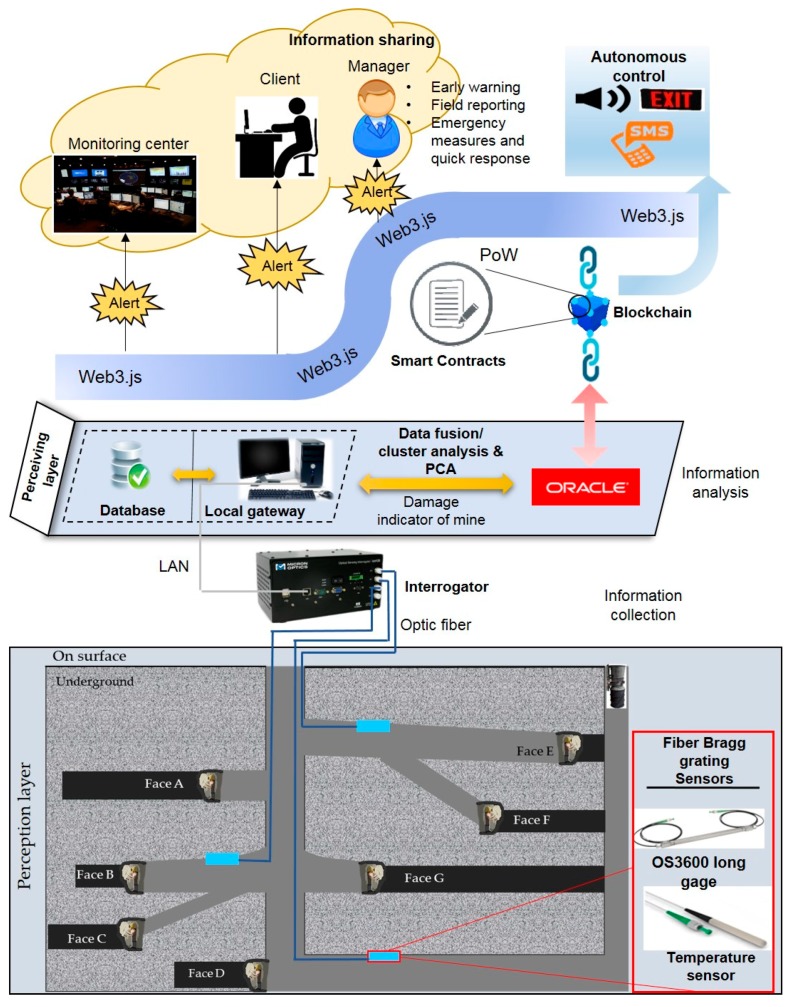
Complete framework of blockchain-IoT network for the use case of an underground coal mine-SHM.

Figure 2 shows the complete architecture of the blockchain-based smart contract for the use case of an underground coal mine SHM.

The present study defines four categories of “undamaged, warning, alarming, and damaged” based on the values of DIM. These categories and DIM values are summarized in Table 1.

### 4.2. Smart Contracts

For the SHM data, the changes in the sensed values cause a change in the DIM value, which can be easily compared with pre-defined threshold limit values in smart contracts. The proposed system is comprised of a main smart contract named Monitoring&Control. It calls a function DIM_Monitor () to compare the value of DIM with the DIM-stored threshold values in the smart contract. The function then calls an object Analyze (). The associated sub-contracts and functions serve as a directory instead of reporting back to the main smart contract and control the attached actuators to alert the managers and monitoring teams. Moreover, the sub-contracts write the transactions in the core blockchain network as a permanent record. However, for simplicity and efficient deployment, all the main and sub-contracts should be written and deployed separately in a blockchain network, calling each other using their addresses. The main contracts with their functions are given in Table 2.

Our proposed network registers each participant as *P_i_* ϵ *P*. This network only accepts the operation from the added participants. Each participant has the authority to either accept or reject the requests and can trace changes in the blockchain network. Participants should have IDs and secret signature keys to join this network and to inquire about the data history. The input algorithm for registering a participant is based on the number of remaining unregistered participants (*No._unreg_*), number of modifications (*No._mod_*), and the hash values of modifications (*dH*), while the outputs are the participant’s public key (*P_PK_*), private key (*P_PVK_*), and addresses (*P_Addr_*) achieved from secret signature keys and public keys. Pseudo-code for participants’ registration is given as shown in Algorithm 2.

**Algorithm 2.** Pseudo-code for registering a participant.**Inputs:** No. of remaining unregistered participants (*No._unreg_*), No. of modifications (*No._mod_*), Hash value set of modifications (*dH*); **Output:** Participant’s public key (*P_PK_*), participant’s private key (*P_PVK_*), participant’s addresses (*P_Addr_*), participant secret key (*P_SK_*)Step 1**For***i* = 0; *i* < *No._unreg_*; *i* + + **do**
Step 2 network generates public key *P_PK_* and private key *P_PVK_* for participant *P_i_* ϵ *P*
Step 3 *P_PK_* generates address *P_Addr_* for *P_i_*
Step 4 network generates a random number *X_i_^u^* ϵ Z_q_^*^ for participant *P_i_*Step 5 network compute *X_i_^S^* = (*X*−*X_i_^u^*) mod *q* and sends (*X_i_^S^, Ui)* to TAStep 6 TA generates a random number *y_i_^u^* ϵ Z_q_^*^ for participant *P_i_*, computes *y_i_^S^* = (*y* − *y_i_^u^*) mod *q* and stores *y_i_^S^*Step 7 Each *P_i_* has its own secret signature key *P_SK_* (*X_i_^u^*, *y_i_^u^*)Step 8
**end for**
Step 9**return***P_PK_*, *P_PVK_*, *P_Addr_*, and *P_SK_*

**Figure 3:**
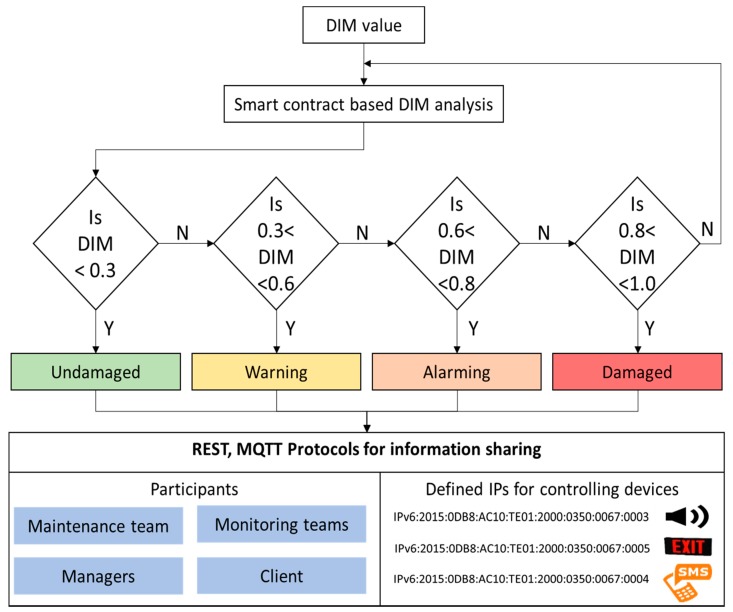
Flow chart of the smart contract and IoT network for transparent information sharing for the selected data of an underground coal mine.

Figure 3 shows the algorithm of the used smart contract based on threshold values of DIM in an underground coal mine for efficient information sharing among all the participants and to trigger an alert in an emergency. For simplicity, we have included demo smart contracts in one file on GitHub [51]. However, for easy replacement the contracts should be stored distinctly on the blockchain, calling the addresses of each other.

### 4.3. Logical Flow of Smart Contracts

**Figure 4:**
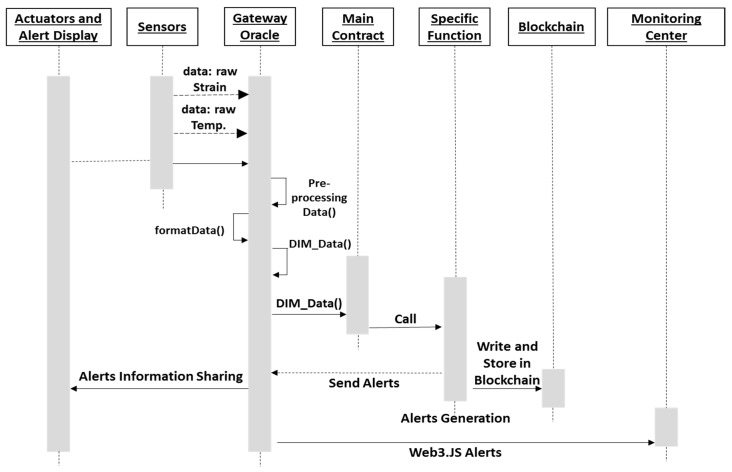
Logical execution flow of proposed system: sensors data is sent to interrogator, and gateway running with Oracle, the gateway implements pre-processingData (), formatData (), and DIM_Data () functions to determine the DIM of the underground mine structure. The DIM determined from sensed data is read by the smart contract to compare with the stored threshold DIM values. Upon meeting the smart contract conditions for the various classes of DIM, the smart contract generates necessary alerts and a transaction will be written to the blockchain.

The logical flow of the proposed smart contract is shown in Figure 4. In this study, smart contracts follow the defined threshold limit values of DIM to respond, control, and generate alerts according to the conditions. The user interface is managed by a decentralized application (DApp) for remote monitoring, and this interface allows the managers to inquire. Managers will have special administrative access to all the data. The information from various sensors is aggregated and formatted in the back-end of the DApp and after pre-processing and proper arrangement it is forwarded to the smart contract connected through Oracle.

## 5. System Analysis

### 5.1. Simulation Setup

All the simulation experiments were performed on an Intel Core i5 CPU 3.90 GHz with 16 GB RAM running on Windows 10. Our proposed blockchain scheme was simulated as a private network using go-Ethereum and the installation of Mist browser enabled distributed blockchain network. Genesis block was defined, and the SHA-256 hash function was deployed. Ethereum testnet debugged and tested our proposed model. To activate blockchain as a service, we deployed Mininet to all the miner’s and edge nodes, so that the timestamped SHM data with its hashes could be placed in it.

For comparison, we also executed our proposed blockchain model in Amazon EC2 cloud with secp256k1 signature code from Ripple. A series of experimental trials were performed to evaluate the performance of the proposed SHM system in terms of scalability, efficiency, and throughput. The experiments were performed considering virtual machines for a maximum number of nodes of 20 because (i) the number of participants in an SHM system is always limited, and (ii) the proposed system showed more stability for fewer than 20 nodes. The proposed network was evaluated from a pre-defined pool of transactions, from which random transactions were called for each participant. Blockchain immutably updates and records the datastore state of each transaction. The results of these simulations are based on the user’s perspective. Since we inherited the SHM of an underground coal mine from our previous work [50], we omit further SHM analysis here.

*Simulation setup:* The throughput trials were performed by sending transactions to the network. These transactions were counted based on the confirmation of receival time. The conditions under which experiments were performed were as follows: number of nodes = 20, acceptance threshold step δ = 0.1, number of requests per s = 1200, and transactions per proposal were set at 1600. Each trial was repeated five times against a varying number of nodes to obtain an average.

### 5.2. Network Performance Evaluation

**Figure 5:**
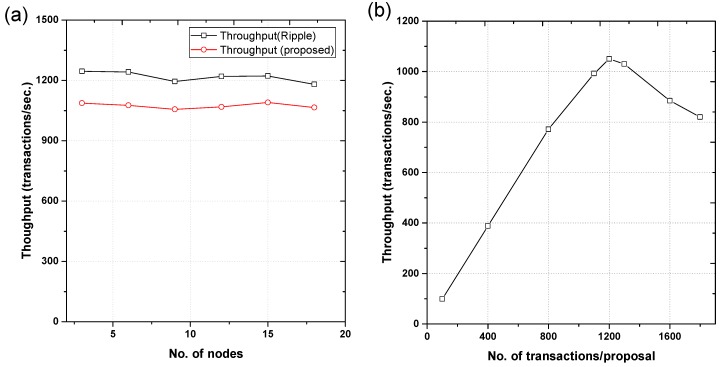
(**a**) Throughput of the proposed blockchain distributed network in comparison to Ripple, and (**b**) Throughput of proposed blockchain architecture for 15 nodes to check the scalability.

*Throughput:* The throughput of the proposed system was determined by varying the number of nodes from 3 to 18 with each increment of 3. Throughput was observed as the number of transactions per second to the number of nodes. Results are shown in Figure 5a. The throughput of the proposed network is comparable with that of Ripple, i.e., 1075 transactions per s. A minute decrease in the throughput of the system was observed with the increase in network size for both Ripple and the proposed system. Decrease in throughput happened when the number of nodes was increased from 15. Therefore, the optimum number of nodes for this proposed network is 15. Overall, compared to Ripple, the proposed system shows greater consistency. It should be noted here that, unlike Ripple, the proposed network is more flexible as it does not deal with financial transactions.

In the second set of experiments, the proposal size was increased to check its effect on the throughput of the network. For these experimental trials, the number of nodes was fixed to 15 and the number of transactions per proposal were varied from 100 to 1800. The results are shown in Figure 5b. The throughput of the proposed network increases until it reaches a peak value of 1050 transactions per second at 1200 transactions per proposal. When the transactions per proposal increased to more than 1200, transaction per second decreased, or the throughput deteriorates. The transactions per proposal dropped to 810 at 1800 transactions per second. Thus, the optimum number of transactions per proposal can be defined as 1200 in the case of our proposed network.

**Figure 6:**
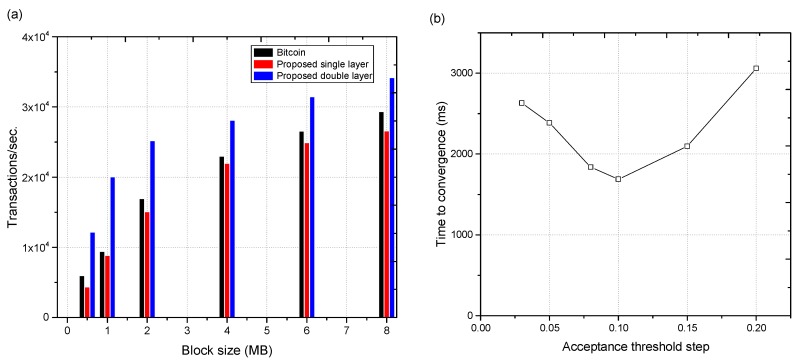
(**a**) Block size against transactions/s of proposed blockchain architecture in comparison with bitcoin and the proposed single layer architecture, and (**b**) convergence time of proposed blockchain architecture against acceptance step δ.

*Effect of block size:* By changing the size of a block, the number of transactions of our proposed system (double layer), proposed system (single blockchain layer), and bitcoin were observed. The results are shown in Figure 6a. These results clearly demonstrate the effect of block size on the number of transactions per second. The data of our simulation is based on the actual values of the bitcoin blockchain [52]. In comparison to bitcoin and the simple blockchain model, our proposed architecture is more efficient (Figure 6a).

*Convergence:* The convergence rate for the proposed system was determined by fixing the number of transactions per second to 1050, the number of nodes to 15, and requests per second to 1200. Figure 6b shows the convergence rate of the proposed system. The least time for convergence was achieved for δ = 0.1. As the values varied from 0.1, the convergence time increased.

**Figure 7:**
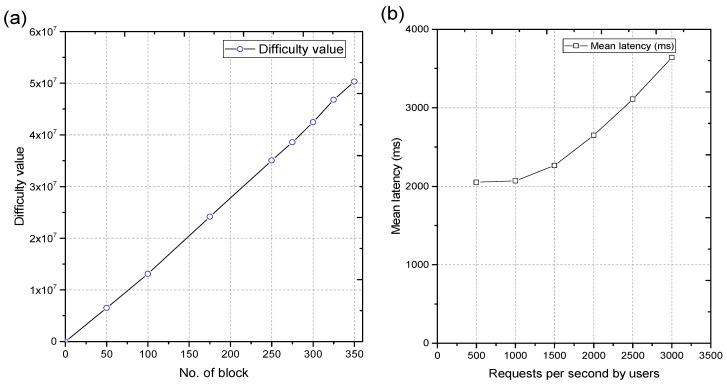
(**a**) Difficulty variation vs number of blocks, and (**b**) mean latency vs. number of user’s requests.

*Difficulty value:* In the blockchain network, PoW consensus mechanism has vital importance, as it applies hard-to-forge computations to solve tasks. Basically, PoW is a tiresome process to solve the puzzle such as a hash collision. Figure 7a depicts the difficulty values against the number of blocks for our proposed network. As the number of blocks increases in a network, the difficulty level increases linearly. Moreover, with the increase of difficulty values, the miner’s nodes need more computation power, and mining time also increases significantly. Therefore, it is imperative to find a compromise between difficulty value and target response time of the system.

*Latency:* Delay in the confirmation of a transaction is known as the latency. Along with throughput, it represents the performance overhead of the architecture. Figure 7b demonstrates the results of latency in milliseconds with the increase of requests per second for 15 nodes. It increases linearly with the increase in the number of requests per second. It also increases with the increase of the number of nodes, as they provide more communication to the network. The overall response of the model remains stable for 15 nodes.

### 5.3. Comparison to Traditional Systems

As a young technology, blockchain is providing society with decentralized solutions. Its applications, such as that proposed in the present study, are quite different from the previously developed centralized SHM systems. Table 3 compares the proposed study with current SHM systems. Most of the previously developed systems are limited to only monitoring and to conventional means of communication and data storage. Few studies have considered cloud computing for SHM, and the architectures of those comparable systems are centralized causing problems with bottlenecks, data security, single point failures, and less reliability for inter-organizational transparent information sharing.

### 5.4. Data Security Analysis

The proposed private and consortium-based blockchain network provides data security in the following ways:

(i) The generation and verification of a new block always requires most numbers of signatures from the authenticated members of networks. Signatures from authenticated members prevent the entrance of an unwanted member in the network and any change or manipulation of SHM data. Thus, such blockchain networks ensure data security.
(ii) As in SHM, the data itself is nothing, but the valuable information extracted from the data has prime importance. Therefore, the present study has been designed to utilize threshold limit value smart contracts. The proposed system provides another layer of data security by simply deploying DIM-based smart contracts for autonomous decision making, instead of placing entire monitoring data in a decentralized network.
(iii) The proposed system only stores the transactions in the form of a ledger. This provides data security for both SHM service providers and the client. A detail record of transactions can be recalled at any time for settling disputes and new design considerations upon the approval of participants.
(iv) Our system provides security against external attacks, as any rogue node can attack the system by submitting an invalid change request. A smart contract will only accept the requests from the pre-identified and authorized participants. All the other requests are simply rejected by the system.

In this case, if an internal advisory submits defective changes, the changes should have a minimum number of approved signatures. Such as attack can then only succeed if the internal advisory controls more than half of the nodes. Moreover, such attacks leave traceable footprints on the blockchain, which are helpful to identify attacks.

## 6. Discussions and Limitations

Our system provides the authenticated and immutable records of SHM with distributed networks. Such blockchain-IoT networks can easily be deployed for the SHM of civil structures. The smart contracts for the SHM of civil structures should follow detailed specifications and standards. However, there are some limitations of the proposed systems:

- The SHM data adopted in this study is from an underground coal mine, taken as an example to demonstrate the feasibility of blockchain-IoT networks and smart contracts for SHM. The smart contract presented here is only for the defined conditions, so it should not be considered as general for all types of structures. For its application in various domains of SHM, smart contracts should first be defined according to the required conditions of structures, which may cause changes in the overall flow of the smart contract.
- In the case of public blockchain networks, the efficiency of PoW is a big question, as it takes too long to place data in a blockchain, which is not acceptable for SHM applications. Therefore, further studies are needed to check the efficiency in the case of public networks.
- For a private blockchain network, it is advisable not to use the same block for all transactions.
- There is no mechanism that can ensure that all the data placed in a blockchain is secure.

Before full-fledged application of distributed blockchain networks for SHM, the network should be properly designed for efficient and autonomous data analysis and decision making according to the requirements.

The proposed network demands that a minimum number of nodes must remain online to verify the blocks’ generation and new transactions. As our proposed architecture is based on Ethereum, the block-time in Ethereum is much faster than bitcoin, and faster block-time may hamper the security of the network by adding numerous stale blocks which are not a part of the main chain but compete in the mining process. For applications such as that proposed in this study, real-time monitoring demands the faster block-time and thus become more open to security risks. In addition to these issues, software vulnerabilities of smart contracts can be exploited by hackers resulting in high risk to organizations, miners, and blockchain network.

## 7. Conclusions

Cross-institutional transparent information sharing and data security with autonomous data analysis and decision making are the big challenges in IoT–SHM. In this context, we have focused on these limitations and proposed a blockchain-based distributed architecture for sustainable SHM. In this work, the PoW consensus mechanism was used to ensure transparency, data security, and data storage. A permissioned and consortium-based blockchain network was established to execute smart contracts. The smart contracts would trigger alerts by following the pre-defined threshold limit values to analyze the streamed data. Ethereum-based simulations were run by placing SHM data. Results showed the proposed network performs well for 15 nodes, 1200 transactions per proposal, and for acceptance threshold step of 0.1. The experimental analysis results showed the effectiveness of the proposed blockchain model for SHM in terms of data security, storage, efficiency, and transparent information sharing among the involved parties.

Blockchain is a young technology and its integration with the IoT domain will revolutionize the world by speeding up the interactions of companies, organizations, governments, and citizens. Leveraging blockchain with conventional IoT–SHM will not only bring closer untrusted parties but also open new business and research horizons. Based on some of the limitations of the proposed network, there is still the room for improvement. Future studies related to adoption of blockchain distributed networks in IoT–SHM should address multiple confirmations along with faster block-time for newly mined blocks to avoid double-spending. Moreover, our future work will include exploring the more detailed implementation of smart contracts integrated with artificial intelligence to enhance confidence in autonomous decision making for SHM.

## Figures and Tables

**Table 1:** Categories of the structural states of an underground coal mine with respect to the damage index of mine (DIM) values.

DIM Value Range	Mine Condition
0–0.3	Undamaged
0.3–0.6	Warning
0.6–0.8	Alarming
0.8–1.0	Damaged

**Table 2:** Types of smart contracts and their functions.

Smart Contracts	Functions
Participants	AddParticipant DeleteParticipant SearchParticipant VerifyParticipant
Monitoring&Control	DIM_Monitor Analyze Generate_Alert Autonomous_IP_communication

**Table 3:** Comparison of functions of the proposed blockchain-IoT network with traditional structural health monitoring (SHM) systems.

Functions and Properties	SHM Studies				
	Simple [53]	WSN [38,54]	IoT [41,42]	IoT-Cloud [19,20]	Proposed
Decentralize	Fully centralized	Fully centralized	Fully centralized	Partially centralized	Fully decentralized
Reliability	Highly unreliable	High data tempering	Data can be tempered easily	Easy data tempering	Transparent and trustworthy inter-organizational information sharing (No tempering, original data)
Data storage, privacy, security, and confidentiality	Low	Low	Medium	Medium	High (access control for participants)
Immutability	No	No	No	Partially	Yes
Real-time	Near-real time	Yes	Yes	Yes	Near-real time
Communication and transparent information sharing	Only limited to monitoring	Limited to monitoring	Monitoring and data processing	Monitoring, data processing, and participant-to-participant (P2P) information sharing	Smart contract-based data analysis for autonomous decision making, participant-to-machine (P2M) and machine-to-machine (M2M) communication
Interoperability	Low	Low	Medium	Medium	High
On-demand maintenance	Low	Low	Medium	Medium	Efficiently high
